# Reviewing the Literature on the Effectiveness of Pressure Relieving Movements

**DOI:** 10.1155/2013/124095

**Published:** 2013-01-13

**Authors:** Rachel Schofield, Alison Porter-Armstrong, May Stinson

**Affiliations:** Rehabilitation Sciences and Research Centre, School of Health Sciences, University of Ulster, Jordanstown, BT37 0QB, UK

## Abstract

Sitting for prolonged periods of time increases seating interface pressures, which is known to increase the risk of developing pressure ulcers. Those at risk of developing pressure ulcers are advised to perform pressure relieving movements such as “pushups” or “forward leans” in order to reduce the duration and magnitude of pressure acting on the vulnerable ischial tuberosity region. The aim of this review was to synthesize and critique the existing literature investigating the effectiveness of pressure relieving movements on seating interface pressures. The twenty-seven articles included in this paper highlight the need for further research investigating the effect of recommended pressure relieving movements on the pressures around the ischial tuberosities. Furthermore, this review found that the majority of individuals at risk of developing pressure ulcers do not adhere with the pressure relieving frequency or magnitude of movements currently recommended, indicating a need for pressure ulcer prevention to be explored further.

## 1. Introduction

Sitting for prolonged periods of time is thought to increase the risk of developing pressure ulcers [1, 2]. Sitting forces the weight of an individual against the supporting seat surface which compresses the soft tissues around the buttock area between the chair and the bony ischial tuberosities. This pressure causes an obstruction of blood flow that when combined with limited movement, poor sensation, malnutrition, and increased age can eventually lead to ulceration [3–5]. These severe, yet usually preventable wounds are relatively common, spanning acute, rehabilitation, and community settings [6, 7], as such, the treatment of pressure ulcers is considered to outweigh the social and financial costs associated with prevention [7].

One of the most effective preventative methods in terms of cost and pressure relief is regular repositioning [8]. Within rehabilitation, individuals at risk of developing pressure ulcers are taught and encouraged to perform regular repositioning movements in order to redistribute the build-up of pressure around the ischial tuberosity and sacral regions. These repositioning movements include vertical pushups, lateral and forward leans. Occupational therapists being responsible for seating and postural care are ideally placed to educate the individual and their carers on good skin health and the importance of relieving pressure at the seating interface regularly [9].

Currently, individuals “at risk” are advised to change their posture by performing pressure relieving movements as often as every 15 to 30 minutes [10, 11]. However, the evidence that these pressure relieving movements effectively redistribute pressures between the individual and the seating surface, known as seating interface, has not been explored. The aim of this review was to synthesize and critique the existing literature on the effectiveness of pressure relieving activities on seating interface pressures.

## 2. Materials and Methods

An electronic search using a range of databases (AMED, CINAHL, Cochrane, Embase, and Medline) was conducted for the years 2002–2012. A combination of keywords was used including seating; sitting; interface pressure; pressure ulcer; decubitus ulcer; activities of daily living; movement; pressure relief; posture; weight shift; reposition; tilt and recline. 

Articles were selected if they were written in English and described experimental research in which functional body movement (both passive and active) was manipulated and seating interface pressures were measured. Studies which did not investigate pressure relieving movements or activities were excluded. No limits were enforced on the type or age of participants included in each study.

**Figure 1:**
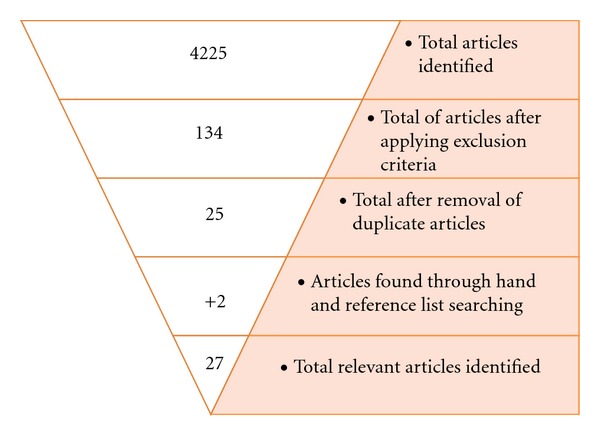
Flow diagram of search strategy.

Initial database searches identified 4225 articles. Application of exclusion criteria reduced the number of articles to 134, which were further reduced to 25 by removing duplicates. An extensive review of the reference list of each included publication and hand searching identified 2 relevant articles. The search procedure identified 27 studies (Figure 1) that met criteria for inclusion in this review summarised in Table 1.

Healthy, able-bodied individuals can sit for lengthy periods without developing pressure-related injuries. Two studies measuring the frequency of seated movements found that able-bodied individuals, with no or little risk of developing pressure ulcers, make considerably more seated postural movements than the rate of seated postural movements recommended by the National Pressure Ulcer Advisory Panel [44].

Linder-Ganz et al. [23] identified that on average, 10 able-bodied participants, seated in a standard wheelchair, changed posture by approximately 15° every 9 ± 6 minutes and 8° every 6 ± 2 minutes in the sagittal and frontal planes, respectively. Unfortunately the value of seated postural movements for relieving or redistributing pressure at the seating interface was not recorded or documented by Linder-Ganz et al. [23]; however, a later study [32] reported that frequent postural movements when seated altered interface pressure and can restore blood flow, thus facilitating tissue health. This study found that 19 healthy able-bodied male subjects changed their posture on average 7.8 ± 5.2 times an hour in the frontal and sagittal planes when sitting in a wheelchair. Additionally, with each posture change, subcutaneous oxygen saturation increased on average by 2.2 ± 2.4% [32].

Despite reporting that seated movements affected interface pressures, Reenalda et al. [32] did not document the actual changes in interface pressure. However, it can be deduced that a decrease in interface pressure occurred during posture shifts which allowed tissue oxygenation and perfusion to occur [3]. 

Although both studies report a favourable outcome for encouraging frequent postural movements to individuals at risk of developing pressure ulcers, their findings are limited to the results of able-bodied individuals, with healthy tissue; therefore, results are not generalisable to the wider population or those most at risk of developing pressure ulcers. Furthermore, it could be argued that the high rate of seated movements reported may have been to alleviate discomfort which is associated with high interface pressures [45] or unease of sitting in a chair of unusual choice for an able-bodied individual and fatigue from sitting over 60 minute [32] to 90 minute [23] period without any lower extremity movement [46]. However, both studies report similar rates of seated postural movements performed by healthy able-bodied individuals, which is in contrast to the rate of pressure relieving movements recommended to [44] and performed by those most at risk [43]. 

Yang et al. [43] demonstrated the infrequency with which 20 community dwelling manual wheelchair users with SCI engaged in pressure relieving movements, by remotely monitoring their daily sitting behaviours over a one-week period. Manual wheelchair users were found to spend an average of 9.2 hours (median 9.7, range 3.2–12.2 hours) per day in their own wheelchair and the average pressure relieving frequency was 9.4 times a day. Considering pressure relieving behaviour was defined as any lift off activity lasting longer than 10 seconds, [43] and as it has been reported that tissue reperfusion rates for this population can take up to 300 seconds [24], the quality of the movements in relation to pressure relief is questionable, thus illustrating the rarity of pressure relieving movements performed. Additionally, the average time of uninterrupted sitting was around 97 minutes (median 62, range 24–284), which indicates that participants in this study did not adhere to pressure relieving recommendations of relieving seated pressures as often as every 15 minutes [44]. 

It is of note that not all of the participants in this study used a pressure redistributing cushion despite the participants having a diagnosis of paraplegia (*n* = 11) or tetraplegia (*n* = 9) which would indicate that they were at increased risk of ulceration. Interestingly, participants with pressure redistributing cushions (*N* = 16) showed a significant increase in uninterrupted sitting time (*P* = 0.029) than those without pressure redistributing cushions (*N* = 4) [46]. Considering that cushions, like human tissue, need a recovery period of off loading to return to their original form before they can be put under stress again highlights the increased level of risk that some of the participants were exposed to by not frequently engaging in pressure relieving movements. 

Similarly, results from postal questionnaires investigating the preventative health behaviours and perceived risk of developing pressure ulcers of community dwelling wheelchair users [39] and persons with spinal cord injury [12] identified a low performance rate of pressure relieving movements. 

Bloemen-Vrencken et al. [12] found that over half of the 410 respondents did not engage in pressure relief movements frequently and only 20.9% of responders always did some type of pressure relief every 30 minutes. Likewise, Stockton and Parker [39] discovered that although 80% of their 136 wheelchair-dependent sample reported they were able to perform weight shifting movements, only 20.8% engaged in pressure relief activity once an hour and a further 54.7% moved less than once per hour, indicating a lack of adherence rather than ability. 

Bloemen-Vrencken et al. [12] found that respondents who had experienced pressure ulcers implemented significantly more pressure ulcer prevention techniques such as relieving pressure regularly than those who had not experienced secondary complications (median total score of 256 respondents with no pressure sores = 45, median total score of 154 respondents having experienced pressure sores in last 12 months = 48, *P* < 0.05). Conversely, Stockton and Parker [39] reported that respondents who had experienced a pressure ulcer did not significantly alter the frequency of pressure relieving movements performed. 

Coggrave and Rose [15] identified ease of performance as the main reason few adhered to recommendations. This small-scale clinical study found that for 46 spinal cord injured participants, the mean duration of pressure relief required to raise tissue oxygen to unloaded levels was 1 minute 51 seconds (range 42 seconds–3 minutes 30 seconds), which may be considered a long time to hold a pressure relieving position [15]. 

### 2.1. Key Message

Despite ability, it has been shown that very few manual wheelchair users adhere to the frequency of pressure reliefs recommended [12, 39, 46]. Policy guidance suggests that wheelchair users should be educated to perform pressure relieving movements regularly and be advised not to sit for longer than 2 hrs in the same position [10]. 

## 3. Tilt and Recline as Pressure Relief Movement

Wheelchair tilt systems passively tilt the whole chair back, while maintaining a constant hip and knee angle. Depending on the extent of the tilt angle applied, wheelchair tilt systems can affect postural control, digestion, and reduce pressure around the vulnerable ischial tuberosity region by redistributing body weight from the seat to the backrest.

Two studies investigating the impact of tilt on blood flow and localised tissue loading for 11 individuals with SCI [35] and the load redistribution qualities of variable position wheelchairs with 6 able-bodied and 10 SCI individuals [37] found that the largest decrease of seating interface pressure occurred during larger tilts of up to 55° from an upright sitting position [35, 37]. As the ischial tuberosities are a curved structure, it may be postulated that a larger movement would be needed to offload the tissue around this area.

However, results are based on small study populations and equipment and seating conditions varied on both studies. Sonenblum and Sprigle [35] used laser Doppler Flowmetry and a custom sensor from FSA (Vista Medical, Winnipeg, Canada) affixed to the skin at the apex of the ischial tuberosity region, whilst participants that sat in their own chair fitted with their prescribed pressure relieving cushion. Using a repeated measures design, each tilt was normalised against each starting position, and by not altering the participants original chair configuration, Sonenblum and Sprigle [35] attempted to create a more realistic interpretation of the effect of tilting on pressure and blood flow for wheelchair users in their “naturalistic” state.

By contrast, Sprigle et al. [37] utilized a single seating system to standardise all the support surfaces and seat and back articulations and placed pressure sensing mats (CONFORMAT 5315QL; TEKSCAN, Boston, MA, USA) below the cushions of the seat and backrest to monitor load redistribution during tilting. However, measuring average pressure over the entire mat may have confounded results, as during a tilt, gravitational forces may have influenced the pressure under the thigh area more than under the ischial area. 

Despite methodological differences it is reassuring that both studies reported reasonably similar results indicating that tilting, as far as the seating system permits, reduced interface pressures.

Compared to tilting, reclining was found to unload the seating interface to a larger degree [37]. Recline increases the seat to back angle of a chair and can necessitate various positions from an upright seated position to a fully reclined supine position. Sprigle et al. [37] found that reclining the backrest fully to 90° produced a 61% reduction on seat forces when compared to upright sitting for both able-bodied and SCI participants. This was to be expected, as lying back to 90° effectively spreads the pressure over a larger surface area, compared to sitting; hence reclining was found to reduce interface pressures [37]. 

Similarly, Stinson et al. [38] found that greater recline angles reduced average seating interface pressures, when investigating the relationship of gender, body mass index, and seating position with seating interface pressures in able-bodied participants. Reclining the chair by 30° was found to significantly reduce average pressure (*P* < 0.001). However, reclining 10°, 20°, and 30° did not significantly alter maximum pressure, which is the highest individual pressure recorded by a sensor over the entire pressure mat. Maximum pressure is considered an unstable measurement [47] as it focuses on the pressure recorded by one single sensor and therefore changes frequently, which may confound results.

Interestingly, armchair recline with feet supported was found to significantly increase average interface pressures [38]. However, results were based on measurements from able-bodied participants, known to have healthier, less atrophied tissue than individuals at risk of developing ulcers. Conversely, wheelchair recline with feet supported was found to stimulate greater unloading in a SCI cohort, who are physiologically predisposed to developing pressure ulcers than in a healthy able-bodied cohort (*P* = 0.003) [37].

As tilt and recline were both found to reduce pressure at the seating interface, it would be reasonable to assume that reducing the angle between the backrest and the seat surface to lower the rear portion of the seat surface (known as squeezing) would increase interface pressure. However, Maurer and Sprigle [26] found no evidence to suggest that “squeezing” a wheelchair frame to induce posterior seat inclination negatively affected seating interface pressures. As squeezing increased, more of the individuals came into contact with the seat surface; however, pressures around the ischial region remained the same thus limiting the value of “squeezing” the chair for relieving interface pressures. 

### 3.1. Key Message

 The studies found that increasing the angle of tilt and increasing recline reduce the loading of pressure at the seating interface. Policy guidelines suggest that education and training in the appropriate use of equipment to facilitate pressure relief should be given to both the individual at risk of developing the pressure ulcer and their caregivers [44].

## 4. Pressure Relief Behaviour of Individuals with Tilt/Recline Systems

Lacoste et al. [21] found that 97.5% of the 40 powered wheelchair users interviewed used their wheelchair daily, of which ≤35% used tilt or recline for physiological functions such as relieving pressure. Many of the respondents used the larger tilts (31–45°) for rest rather than pressure relief [21] questioning the practicalities of performing such a large movement regularly. Additionally, none of the respondents had a wheelchair that tilted beyond 45°, possibly explaining why among the few subjects who used their seating system for pressure relief, ≥50% used small amplitudes (≤15°) and 40–50% used medium amplitudes (16–30°) of tilt. However, Lacoste et al. [21] based their findings on the subjective opinion of 40 powered wheelchair users and recall bias and perception of illustrated seat angles may have clouded the accuracy of the findings reported. 

Conversely, three studies, though methodologically different, also found that few subjects tilted past 45° [16, 33, 36], despite all [33] or nearly all [16, 36] of the participants using a wheelchair that was capable of at least a 40° tilt.

Ding et al. [16] reported that over a 2-week period, 11 subjects occupied their wheelchairs for 11.8 ± 3.4 hours, transferred in and out of their wheelchairs 5 ± 5.3 times, and performed 19 ± 14 tilts on average per day. Similarly, Sonenblum and Sprigle [36] and Sonenblum et al. [33] found that power wheelchair users occupied their wheelchairs an average of 11 hours [33] and 11.7 hours per day [36] and performed tilts 3.0 ± 2.9 times [36] and 4.3 ± 3.9 times per occupancy hour [33].

However, a change in tilt was classified as an angle change of ≥2.5° that lasted for at least 1 minute [16] or any changes of more than 5° held for more than 20 seconds [33, 36]. The definition of a tilt or recline involving a minimal change in backrest angle may have overinflated results reported. However, Ding et al. [16] used an algorithm to filter raw data and reduce the likelihood of accidental fluctuations in backrest angle interfering with results. 

Tilts and reclines of less than 20° were found to be more frequent and held for a longer duration of time than larger tilts and reclines [16], though 2 of the 11 wheelchairs used in this study did not tilt past 20°, which may have skewed results. Similar findings were reported by Sonenblum and Sprigle [36] and Sonenblum et al. [33] as the subjects in these studies spent the majority of time in tilt angles <20°, despite all [33] or nearly all [36] of the participants chairs having the capability of tilting to or past 45°. However, although participants were found to tilt relatively frequently, these tilts, according to the literature, were not large enough to relieve pressure around the vulnerable ischial tuberosity region [37, 38].

### 4.1. Key Message

The studies indicate that despite education, powered wheelchair users do not use the recommended magnitude of tilt or recline required to adequately relieve pressure around the vulnerable ischial tuberosity region. Interestingly, these larger magnitudes of tilt and recline were used to relieve pain and discomfort. Policy guidelines exemplify that although comfort is of primary importance, a flexible repositioning schedule based on the individual's preferences and tolerance should be encouraged as variable position seating is reported to redistribute pressures from around the vulnerable ischial region [44]. 

## 5. Seat Adjustments to Induce Postural Movements

Moes [27] explored the relationships between interface pressure, pelvic rotation, and body characteristics of 19 able-bodied participants. Through multiple regression techniques it was shown that intrinsic characteristics including ectomorphic index, gender, mass, and the body anthropometry greatly affected pressures at the seating interface during forward and backward rotations of the pelvis. 

Similarly, Vos et al. [42] investigated postural and chair design effects on seating interface pressures using 12 ergonomic office chairs and 24 able-bodied participants (12 male; 12 female). They found that the greatest impacts on seating interface pressures were chair design differences, followed by participant characteristics such as gender, and lastly postural movements. 

Both studies investigated a relatively small postural change in pelvic tilt [27] and pelvic and trunk tilt [42] which are known to have a minimal effect on interface pressures [37, 38]. However, the slight pelvic movement was enough to effectively reduce the maximum pressure for a typically ectomorph (thin) person [27]. Similarly, Vos et al. [42] found that an increased posterior pelvic tilt significantly reduced pressure values for all participants.

Conversely, posterior pelvic tilt was found to have no significant effect on maximum pressure for 10 healthy male participants [20] and actually statistically increased the estimated shear force on the seat (*P* < 0.01). Shear force is the distortion of a body by two oppositely directed parallel forces; hence, such forces can damage skin and are known to contribute to pressure ulceration [44]. 

Kobara et al. [20] measured pressure distribution while each subject sat in the same chair, at various distances from the backrest and leant back, thus simulating a posterior pelvic tilt. The results showed that the locations of the points of maximum pressure were significantly displaced forward in all positions, upright (*P* = 0.005); sitting 5 cms from the backrest (*P* = 0.008); sitting 10 cm from the backrest (*P* = 0.285). This illustrated the element of shear in all positions and increasing posterior tilt which would increase the risk of pressure ulceration at these sites. Maximum pressure, as explained previously, is considered an unstable measure [47] and was used in this study to gauge the movement of the ischial tuberosities and not to investigate the redistribution of pressure during a pelvic movement. Additionally this study used a small able-bodied cohort and a chair with a hard surface with no alterations for subjects of varying morphologies, which may have confounded results.

Similar to Kobara et al. [20], Van Gefen et al. [40, 41] found that posterior pelvic tilt had a minimal effect on ischial pressures when investigating an experimental simulator chair that could independently control the orientation of the trunk, pelvis, and thighs with 18 healthy male subjects. It is possible that the exclusion of trunk movements limited the degree of posterior pelvic tilt, causing the ischial tuberosities to roll within their original area of high pressure and therefore minimally affecting overall pressure measurements. 

Additionally, Van Geffen et al. [41] reported that a pelvic side elevation of 9° reduced ischial pressure by 34%, which was to be expected as the majority of body weight and therefore interface pressure were redistributed to the lower side of the chair surface. In other words, forcing a pelvic obliquity reduced pressure under the higher ischial tuberosity and undoubtedly increased pressure under the lower weight bearing side. This highlights the increased risk of ulceration individuals with a pelvic obliquity face and the importance of correct positioning and specialist seating prescription. 

Makhsous et al. [24, 25] also evaluated the effect of chair design on interface pressures by periodically removing the ischial support area of a chair every 10 minutes for 10 minutes, compared to traditional pressure relieving movements (push up held for as long as possible or hoisting out of wheelchair for 60 seconds) every 20 minutes over two 1-hour sitting periods using 60 participants (20 paraplegic; 20 tetraplegic; 20 able bodied). 

Both studies reported that removal of the ischial support area of the chair significantly diminished interface pressures around this area and that a significant proportion of the buttock pressure was redistributed to the thigh area during these simulated pressure reliefs. Furthermore, Makhsous et al. [24] noted that of those who could perform push up pressure reliefs, the average push up time achieved was 49 ± 2.8 seconds; however, it took in the range of 200–300 seconds for tissue perfusion recovery to occur. Therefore, participants in this study were unable to hold the pressure relieving movements for long enough to allow tissue reperfusion to occur.

However, comparing a physical pressure relieving movement to a mechanical unloading of the ischial area for 10 minutes (600 seconds) may have skewed results in favour of the adjustable chair. Also, it is possible that removal of ischial support would cause an increase of pressure acting on the upper thigh area in contact with the remaining seat edge. Finally, the ease of removing the ischial section of the seat and the participant's views and dignity are not discussed; hence, further investigation is needed before definitive conclusions are made.

### 5.1. Key Message

These studies emphasise that larger postural movements are more effective in redistributing seating interface pressures. Policy guidelines recommend that clinicians should consider body size, body posture, mobility, lifestyle, and deformity when prescribing seating for individuals at risk of pressure ulceration [44]; hence, poor postural stability and mobility, for example, may indicate the need for a powered wheelchair device to access postures required for adequate pressure relief. 

## 6. Activity to Encourage Pressure Relieving Movements

Postural stability is a prerequisite for the performance of seated movements and activities. Standing and seated stability are widely accepted to be measured by centre of pressure displacement [17–19, 22, 30, 31]. The centre of pressure (COP) is the average location of the pressure, which has the potential to move as a person moves and adopts different positions. 

Four studies considered the reliability and appropriateness of using pressure measurements in the form of COP for assessing seated static and dynamic stability with 12 healthy older adults [19]; 13 children aged 7–15 years [22]; 45 children aged 4–15 years [30]; 42 motor incomplete SCI adults and 10 healthy able-bodied adults [17]. 

Though studying different populations three of the studies employed a multidirectional reaching activity to investigate the repeatability of measuring COP for seating stability [17, 19, 30]. Kerr and Eng [19] measured COP displacement during multidirectional reaches with and without participants' feet being supported; Field-Fote and Ray [17] instructed participants to reach as far as possible with their right hand, while Olsson et al. [30] devised a fictional game where participants pretended to be an aeroplane with both arms out stretched to either side, then reached 1.5 × the length of their arm (from 7th cervical vertebra to the styloid process on the wrist) in each direction. 

Lacoste et al. [22], on the other hand, instructed participants to reach 5 times to a target normalised to each participant's arm length (measured from acromion process to the tip of the middle finger × 130%) in a forward direction and then to the side, to compare the static and dynamic measurements recorded by the Force Sensory Array pressure mapping system and a force platform (AMTI OR6-7). Fortunately, Lacoste et al. [22] found the pressure mapping system to be as effective as the force platform in detecting COP displacement; hence, results of all four studies can be discussed together [17, 19, 22, 30].

Despite methodological and population differences, all four studies found the deviation of centre of pressure to be greater in the forward/backward movement than laterally [17, 19, 22, 30] and test re- test reliability was high for all directions of reach [17, 19, 22]. Greater COP displacement in the forward/backward direction was to be expected, as generally the base of support is larger in this direction (length of the thighs) compared to laterally (width of the hips).

Interestingly, Kerr and Eng [19] found that when reaching in the lateral and backwards direction, foot support significantly reduced COP displacements by 20%; however, in contrast foot support increased COP displacement in the forward direction by 70%. These phenomena may be explained by the supported feet extending the base of support in the forward direction, and limiting the counterbalancing effect of the feet when reaching laterally or backward. 

Parkinson et al. [31] investigated the effect of having no foot support on the COP excursion capability of 38 able-bodied adults (age range from 21 to 74 years) and found that in forward, backward, and lateral reaching tasks, participants chose to swing their legs in the opposite direction of the reach. These results emphasise the counter balancing effect of the lower legs and feet during reaching tasks for able-bodied subjects and question the arc of reach accessible by those with limited lower limb function as the counter balancing effect of the lower limbs may be compromised. 

Karatas et al. [18] found that the COP displacements for 16 SCI participants were significantly smaller in all directions (forward, backward, left, and right) than the measurements for 18 able-bodied subjects. The change in COP was expected to be smaller for the SCI cohort due to physiological differences in SCI individuals. However, it is possible that the able-bodied participants' ability to transfer weight through their lower limbs may have affected results. 

 Karatas et al. [18] found that SCI participants, with a history of pressure ulcers, had a smaller COP displacement than SCI participants with no history of pressure ulcers, which could not be explained by level of injury [18]. Therefore, it is possible that the ability to perform larger postural movements allows for more pressure relief and thus the group of SCI participants who could perform these movements had healthier tissue; however, as other interfacial pressures (such as peak pressure index, total contact area, and dispersion index) were not reported it is unwise to make a judgement.

### 6.1. Key Message

In sitting, COP indicates the degree of upper body movement available. These studies suggest that the greater the upper body movement, the greater the COP displacement and highlight the possibility that the pressure at the seating interface may also be affected. For able-bodied subjects, these studies found that the orientation of the feet in relation to the body impact on the degree of COP displacement available. Policy guidelines; however, for individuals at risk of pressure ulceration suggest that feet should be supported in sitting [44]. 

## 7. Implications for Practice

A review of the literature on the effectiveness of pressure relieving movements on seating interface pressures has shown that the majority of wheelchair users do not adhere with the recommended pressure relieving frequency or magnitude, even when they possess the ability to either physically or passively redistribute their body weight and hence reduce seating interface pressures and the likelihood of pressure ulcers occurring. Therefore, further research into the reasons behind the lack of concordance with pressure relieving recommendations amongst populations at risk should be explored. It is also recommended that more robust research into the effect of seated movements on interface pressures with populations at risk should be further investigated. 

Until more detailed research is carried out, practitioners should continue to follow the current guidelines on pressure ulcer prevention such as the NICE “Pressure Ulcer Prevention and Treatment” guidelines [48] or the National and European Pressure Ulcer Advisory Panel guidelines [44] which both emphasise that practitioners should encourage clients to pressure relieve as regularly as possible.

## 8. Conclusion

None of the studies investigating functional activity and seating interface pressures explored the impact of these postural movements on the pressure around the vulnerable ischial region. Therefore, although it can be ascertained that functional activity influences the pressure at the seating interface, the positive or negative implications of such movements on seating interface pressures are currently not known.

This is particularly concerning as pressure ulcers are most likely to develop around the bony ischial tuberosity region and as the effect of activities on the pressure around this area is unknown, the performance of certain seated activities may aggravate the development of pressure ulcers for populations at most risk of developing such wounds. Hence further investigation into the effect of seated activities on interface pressures is necessary.

## Figures and Tables

**Table 1:** Data extraction table.

Author	Aim	Design	Sample	Activity	Chair type	Interface pressure measurement	Outcome measures
Bloemen-Vrencken et al., 2007 [12]	To describe the health behaviour of persons with spinal cord injury(SC I) living in the community and the relationships between health behaviour, respondent/injury characteristics, and health-related variables	Postal survey	*N* = 410	Questionnaire			The Spinal Cord Injury Lifestyle Scale (SCILS) [13]. The General Health scale of the SF-36 [14]. The Dutch questionnaire version of the Barthel Index (Post et al., 1995)

Coggrave and Rose, 2003 [15]	To explore if a traditional pressure relief of 15–30 seconds was an effective pressure relief	Retrospective review	*N* = 46	Tissue oxygenation measured in sitting position (loaded) and during pressure relief (unloaded) to investigate the duration of pressure relief required for loaded TcPO2 to recover to unloaded levels	Participants' own wheelchair	The Oxford Pressure Monitor, (Talley Group Ltd)	Time spent in pressure relief; Transcutaneous oxygen tension

Ding et al., 2008 [16]	To examine how individuals used powered wheelchair functions during typical ADL	Case series	*N* = 11	Remote monitoring of daily activity 1-2 weeks	Participants' own wheelchair with pressure relieving cushion	Seating Function Data Logger (Ding et al., 2008 [16]) Force Sensory Array Pressure mapping system (Vista Medical, Winneipeg, Canada)	Seating function usage; occupancy time; frequency of tilt, recline

Field-Fote and Ray, 2010 [17]	To investigate the relationships between the seated reach test, trunk excursion, and COP excursion	Repeated measures	*N* = 42 (10 AB)	Reach with left arm extended to the front, left, right, and back	A Kisler platform with a 0.64 cm padded cover	A force platform (Kisler Instrument Corp., Amherst, NY, USA)	COP excursion; relationship between wrist and trunk excursion; relationship between limits of stability and COP excursion

Karataş et al., 2008 [18]	To evaluate COP displacement in SCI patients	Repeated measures	*N* = 34 (18 AB)	Maximum unsupported leaning forward, backward, and laterally	Hard chair no backrest	Pliance seat sensor system (Pliance 16-P Mat, Novel, Munich, Germany)	COP displacement

Kerr and Eng, 2002 [19]	To quantify the limits of stability during a reaching task; determine the effect of foot support; reliability of using COP to test stability	Test retest reliability	*N* = 12 AB	Seated reaching (forward, backward, and laterally)	Rigid platform	Force plate	COP displacement COP velocity

Kobara et al., 2008 [20]	To investigate the relation between the position of the pelvis while sitting in a chair, pressure and shear force	Repeated measures	*N* = 10 AB	Participants sat on chair at various distances from the backrest then leant back to induce a posterior pelvic tilt	Chair with 5 cm foam cushion on seat and backrest	Pressure distribution measure (NITTA Corp. BIG MAT)	Maximum pressure displacement

Lacoste et al., 2003 [21]	To characterize the use of powered tilt and recline systems	Questionnaire	*N* = 40	Questionnaire			Self assessment of comfort/discomfort; rest; posture; functional independence; psychological functions of powered wheelchair

Lacoste et al., 2006 [22]	To establish the validity and reliability of a pressure mapping system to measure seated postural control in children	Concurrent validity and reliability	*N* = 13 AB	Reach forward and to the right and press a button set 130% × arm length (acromion process to tip of middle finger)	Simulator chair	Force Sensory Array Pressure mapping system (Vista Medical, Winneipeg, Canada) Force plate (AMTI OR6-7)	COP displacement

Linder-Ganz et al., 2007 [23]	To measure the frequency of postural changes among healthy subjects sitting in a wheelchair, and the extent of trunk motion during postural changes	Repeated measures	*N* = 10 AB	90-minute sitting period watching movies	A standard wheelchair (“Patriot”, Invacare Co. USA). No additional cushions. No special adjustments	Pressure sensors (Flexiforce, Tekscan Co. MA, USA)	Movements performed in the sagittal and frontal planes

Makhsous et al., 2007 [24]	To investigate the relieving effect on interface pressure of an alternate sitting protocol involving a sitting posture that reduces ischial support	Repeated measures	*N* = 60 (20 AB)	Two 1 hr protocols. Normal sitting with wheelchair push ups once every 20 minutes. Normal sitting and support over ischial area removed every 10 minutes	Intelligent Pressure Ulcer Prevention Cushion seating system	Xsensor pressure mapping system (Xsensor Technology Corp., Calgary, Canada)	Total contact area; average pressure; maximum pressure;

Makhsous et al., 2007 [25]	To study the effect on tissue perfusion of relieving interface pressure using standard wheelchair push ups compared with a mechanical automated dynamic pressure relief system	Repeated measures	*N* = 60 (20 AB)	Two 1 hr protocols. Normal sitting with wheelchair push ups once every 20 minutes. Normal sitting and support over ischial area removed every 10 minutes	Intelligent Pressure Ulcer Prevention Cushion seating system	Xsensor pressure mapping system (Xsensor Technology Corp., Calgary, Canada)	Total contact area; average pressure; maximum pressure; tissue perfusion

Maurer and Sprigle, 2004 [26]	To examine the effect of increasing posterior seat inclination on seating interface pressures	Repeated measures	*N* = 14	Sitting in the simulator chair in four different angles of seat inclination	KISS seat simulator (Invacare Corp, Elyria, OH)	Force Sensory Array Pressure mapping system (Vista Medical, Winneipeg, Canada)	Total force; contact area; peak pressure index; dispersion index

Moes, 2007 [27]	To investigate the variation in sitting pressure distribution and location of the points of maximum pressure with rotation of the pelvis, gender, and body characteristics	Repeated measures	*N* = 20	Forward and backward rotation of the pelvis	Kistler platform	A pressure measuring device [28]. A mirror box [29]. A small antenna attached to the sacrum	Pelvis rotation; location of the points of maximum pressure; pressure distribution; pelvic angle

Olsson et al., 2008 [30]	To investigate whether pressure mapping can be used as a compliment in sitting analysis and to study the test retest reliability of the measurements	Concurrent validity and reliability	*N* = 45 AB	Reach 1.5 × arm length to either side and forward	Wooden Bench	Force Sensory Array Pressure mapping system (Vista Medical, Winneipeg, Canada)	COP displacement

Parkinson et al., 2006 [31]	To obtain normative data on COP excursion capability for lateral reaches	Repeated measures	*N* = 38 AB	Maximal lateral reaches	Rigid platform	Xsensor pressure sensing mat (Xsensor, Technology Corp., Calgary, Canada). Force plate (OR6-5-1, AMTI, Watertown, MA, USA)	Maximum COP excursion capability

Reenalda et al., 2009 [32]	To describe healthy dynamic sitting behaviour and investigate the effects of sitting behaviour on subcutaneous tissue oxygenation	Cross-sectional study	*N* = 19 AB	60-minute sitting period watching TV	Wheelchair	Tekscan CONFORMat pressure mapping device (Tekscan Inc, Boston, MA, USA)	Frequency of posture shifts and impact on subcutaneous tissue oxygenation

Sonenblum et al., 2009 [33]	To monitor and describe the use of powered wheelchairs in everyday life and record the frequency of pressure relieving movements	Case series	*N* = 16	Remote monitoring over 1-2 weeks	Participants' own wheelchair with pressure relieving cushion	The Wheelchair Activity Monitoring Instrument [34]. Uniaxial accelerometer (VTI Technologies, Finland)	Daily wheelchair occupancy; typical position; time spent in small, medium and large tilt; tilt frequency and pressure relieving frequency

Sonenblum and Sprigle, 2011 [35]	To evaluate the biomechanical responses to full body tilt in persons with SCI	Repeated measures	*N* = 11	Sitting in wheelchair at various degrees of tilt	Participants' own wheelchair	A custom sensor (Vista Medical, Winneipeg, Canada)	Average pressure Maximum pressure

Sonenblum and Sprigle, 2011 [36]	To characterise the use of powered wheelchairs	Case series	*N* = 45	Remote monitoring over 1-2 weeks	Participants' own wheelchair with pressure relieving cushion	The Wheelchair Activity Monitoring Instrument [34]. Uni axial accelerometer (VTI Technologies, Finland)	Daily wheelchair occupancy; typical position

Sprigle et al., 2010 [37]	To provide quantitative information on the magnitudes of loading on the body across clinical ranges of tilt, recline, and standing	Repeated measures	*N* = 16 (6 AB)	Sitting in wheelchair in various degrees of tilt, recline, and standing	Levo combi power wheelchair (Levo USA, Brooklyn Park, MN, USA)	Tekscan Pressure Sensor System (CONFORMAT 5315QL; TEKSCAN, Boston, MA, USA)	Rates of unloading and maximum loading

Stinson et al., 2003 [38]	To investigate the relationship between interface pressure and gender, BMI, and seating positions	Group design	*N* = 63 AB	Sitting in armchair set at 10°, 20°, and 30° recline with and without foot support	Armchair	Force Sensory Array Pressure mapping system (Vista Medical, Winneipeg, Canada)	Average pressure Maximum pressure

Stockton and Parker, 2002 [39]	To provide an insight into wheelchair users preventative health behaviours/ pressure relief behaviours	Postal questionnaire	*N* = 136	Questionnaire			Postal Questionnaire: self assessment of physical capability, past frequency of pressure relieving movements, perceived risk, attribution of responsibility for the performance of pressure relief, and other preventative behaviours

Van Geffen et al., 2008 [40]	To investigate the effects of postural adjustments on seat reaction load	Repeated measures	*N* = 18 AB	Participants sat in simulator chair which passively moved their pelvis posteriorly and reclined back	Adjustable simulator chair	Tekscan Pressure Sensor System (CONFORMAT 5315QL; TEKSCAN, Boston, MA, USA)	Average pressure (ischial and sacral) and centre of pressure

Van Geffen et al., 2009 [41]	To investigate the effects of decoupled pelvis rotation on seating interface loads	Repeated measures	*N* = 18 AB	Participants sat in simulator chair which passively moved their pelvis posteriorly and side to side	Adjustable simulator chair	Tekscan Pressure Sensor System (CONFORMAT 5315QL; TEKSCAN, Boston, MA)	Pelvis orientation; seat reaction force; centre of pressure; peak pressure index and sacral load

Vos et al., 2006 [42]	To investigate personal, postural and design factors upon seating interface pressures	Repeated measures	*N* = 24	Each participant sat in each of the chairs	12 ergonomic office chairs	Xsensor pressure mapping system (Xsensor Technology Corp., Calgary, Canada)	Average and maximum pressure

Yang et al., 2009 [43]	To describe the sitting behaviours in community-dwelling manual wheelchair users (MWUs) with spinal cord injury (SCI)	Case series study	*N* = 20	1 week normal activities of daily living	Participants' own wheelchairs	6 force sensor resistors (interlink electronics, Camarillo, CA, USA)	Lift off frequency Cumulative sitting time
